# Inquiry for a Remedy in Cases of Ringworm

**Published:** 1811-07

**Authors:** 


*To the Editors of the Medical and Physical Journal.*

*Inquiry for a Remedy in cases of Ringworm.*

GENTLEMEN,

THE complaint known by the name of Ring-worm, which infests the schools in the neighbourhood of the metropolis, may truly be deemed an *opprobrium medicorum*, from the difficulty with which it is kept under, and its being scarcely ever cured, however various the practice of the different medical men in this metropolis; whilst Tinea capitis, a complaint almost equally common, is more easily mastered. I venture, therefore, to ask any of your intelligent correspondents, if they have ever been able to discover a remedy producing permanently good effects in cases of Ring-worm? The communication of it to the public would relieve many families and practitioners from much disquietude. Though an old practitioner, I am free to confess that I am unacquainted with the history and description of that peculiar affection, in any nosological work.

I am, Gentlemen,
Your humble Servant,
PHILANTHROPOS.

